# Effects of Preoperative Chronic Steroid Use on Postoperative Outcomes in Orthopedic Surgery: A Systematic Review and Meta-Analysis

**DOI:** 10.3390/ph16091328

**Published:** 2023-09-20

**Authors:** Yu-Ting Hung, Wei-Kai Hung, Ching-Chi Chi

**Affiliations:** 1 Department of Anesthesiology, Chang Gung Memorial Hospital, Linkou, Taoyuan 33305, Taiwan; joycehong05.tw@gmail.com; 2 Department of Dermatology, Chang Gung Memorial Hospital, Linkou, 5, Fuxing St., Guishan Dist., Taoyuan 33305, Taiwan; kaixtranquil@gmail.com; 3 School of Medicine, College of Medicine, Chang Gung University, Taoyuan 33302, Taiwan

**Keywords:** corticosteroids, meta-analysis, orthopedic, surgical outcomes, systematic review

## Abstract

Higher rates of postoperative complications have been found in preoperative chronic steroid users. However, the effects of preoperative chronic steroid use on outcomes in orthopedic surgery were unclear. We performed a systematic review of cohort studies examining the effects of chronic steroid use on postoperative outcomes following orthopedic surgery and searched PubMed, Embase, and CENTRAL through 29 April 2023. We included 17 studies with 1,546,562 patients. No increase in 30-day mortality (adjusted odds ratio (aOR) 1.40, 95% confidence interval (CI) 0.64–3.09) and composite thromboembolic events (aOR 1.61, 95% CI 0.99–2.63) but increases in 30-day overall complications (aOR 1.42, 95% CI 1.16–1.75), wound dehiscence (aOR 2.91, 95% CI 1.49–5.66), infectious complications (any infection (aOR 1.61, 95% CI 1.44–1.80), sepsis (aOR 2.07, 95% CI 1.34–3.21), superficial surgical site infection (SSI) (aOR 1.73, 95% CI 1.03–2.89) and deep SSI (aOR 1.96, 95% CI 1.26–3.05)), re-admission (aOR 1.62, 95% CI 1.48–1.77), both 30-day (aOR 1.28, 95% CI 1.03–1.59) and 1-year re-operation (aOR 1.78, 95% CI 1.09–2.92), pulmonary embolism (aOR 5.94, 95% CI 1.52–23.29), and deep vein thrombosis (aOR 2.07, 95% CI 1.24–3.46) were detected in preoperative steroid users. An increased risk of adverse outcomes following orthopedic surgery in chronic steroid users was found.

## 1. Introduction

A systemic corticosteroid is increasingly administered for a wide range of inflammatory and autoimmune diseases, with asthma, chronic obstructive pulmonary disease, and various types of dermatoses being the most common indications. Notably, between January 1989 and December 2008, there was a substantial 34% increase in the prescription of long-term oral corticosteroids in the United Kingdom [1]. Though systemic steroids are beneficial for these diseases because of their anti-inflammatory and immunomodulating effects, chronic steroid use demonstrates some notorious side effects across multiple organ systems [2]. One of the major concerns with chronic steroid use is the adverse effects on the cardiovascular system caused by fluid retention and electrolyte imbalance, which may eventually increase the risk of hypertension and congestive heart failure [3]. Furthermore, the development of a cushingoid appearance is another well-established side effect. Individuals affected by this side effect typically exhibit distinct features, including truncal obesity, moon facies, and a buffalo hump [4]. Furthermore, hypophyseal pituitary adrenal axis suppression can also occur because of the excessive steroids in circulation [2]. The exogenous steroids lead to a negative feedback loop affecting both the hypothalamus and the pituitary gland. Consequently, the production of corticotropin-releasing hormone from the hypothalamus and corticotropin/adrenocorticotropic hormone from the pituitary gland decreases, which results in decreased cortisol secretion from the adrenal cortex [4].

For surgeons and anesthesiologists, there may be increased concerns regarding other side effects of chronic steroid use impacting the immune, musculoskeletal, and cutaneous systems. The immune system is suppressed, and the natural wound-healing process is inhibited under prolonged steroid use, which may predispose patients to undesirable outcomes such as wound infections and wound disruptions [4,5,6,7]. Within the musculoskeletal system, one of the primary concerns associated with prolonged steroid use is the development of osteoporosis [2]. Prolonged steroid use results in reduced calcium absorption in the intestines and increased calcium excretion in the urine. Consequently, the lower calcium level in circulation triggers the production of parathyroid hormone, which, in turn, stimulates osteoclast activity, leading to accelerated bone resorption [4]. Furthermore, steroids also exert an inhibitory effect on osteoblast activity, which adversely affects the formation of trabecular bone [4]. The presence of osteoporosis may pose an additional challenge for orthopedic surgeons while performing surgery [8].

Some studies have investigated the effects of preoperative chronic steroid use in patients receiving surgery and reported a higher rate of postoperative complications, including wound infection [9,10,11], wound dehiscence [9,11], venous thromboembolism (VTE) [9], re-admission [9], re-operation [9], and even mortality [9,11]. However, those studies did not specify the types of surgery performed, which may be the main source of clinical heterogeneity. Recently, many studies focusing on the effects of preoperative steroid use in patients undergoing orthopedic surgery have been conducted [12,13,14,15]. These studies covered common types of orthopedic surgeries and reported a wide range of different postoperative outcomes. However, the results of different postoperative outcomes were inconsistent. The exact risk profile of preoperative steroid use in patients undergoing orthopedic surgeries remained unclear.

We hypothesized that preoperative chronic steroid use adversely affects various postoperative outcomes in orthopedic surgery. The aim of this study was to systematically examine the effects of preoperative chronic steroid use on postoperative outcomes in patients undergoing orthopedic surgery.

## 2. Materials and Methods

This systematic review and meta-analysis followed the Meta-analysis of Observational Studies in Epidemiology (MOOSE) reporting guidelines [16], and the protocol was registered and published on the International Prospective Register of Systematic Reviews (PROSPERO identifier: CRD42022303673). As no identifying information about personal details was shown in this study, ethical approval and informed consent were not needed.

### 2.1. Search Strategies

We searched PubMed, Embase, and the Cochrane Central Register of Controlled Trials (CENTRAL) from their respective inception to 29 April 2023, for relevant studies. The search terms included “preoperative chronic steroids”, “surgery”, and their synonyms (see Table S1). Neither language nor geographic restrictions were precluded. The references of included articles were scanned to check for relevant studies.

### 2.2. Study Selection and Eligibility Criteria

The first two authors independently screened the titles and abstracts to select potentially eligible articles based on the following inclusion criteria: (1) cohort studies, (2) studies consisting of an exposed group of preoperative chronic steroid users and a control group not using any preoperative steroid before receiving orthopedic surgery, and (3) studies reporting surgical outcomes of interest. Our primary outcome was postoperative mortality. Our secondary outcomes included overall complications, wound dehiscence, infectious complications, thromboembolism events, re-admission, and re-operation. Studies with a limited course of steroids (less than 7 to 10 days) and studies under cross-sectional and case-control design were excluded. The full text of potential studies was examined for confirming eligibility. Discrepancies in the selection of studies were handled by discussion with the third senior author.

### 2.3. Data Extraction and Risk of Bias Assessment

We extracted the following data: author, publication year, database, study period, number of each patient group, surgical procedures, codes for identifying the surgical procedures, reported outcomes of interest, and length of follow-up. The number of events and non-events for each outcome was also extracted to calculate the crude odds ratios (ORs) with 95% confidence intervals (CIs). For studies that provide adjusted odds ratios (aORs) derived from multiple regression modeling or matching cohorts, we extracted the aORs they presented.

The risk of bias in each study was evaluated according to the Newcastle–Ottawa Scale (NOS) [17] by the same first two authors, who were blinded to each other’s assessment. Any disputes were arbitrated by consulting the third experienced author.

### 2.4. Statistical Analysis

We performed a meta-analysis using the Review Manager version 5.4 (The Cochrane Collaboration, 2020). The random-effects model was employed because considerable clinical heterogeneity was anticipated [18]. Only studies that provided adjusted risk estimates were included in our meta-analysis for mitigating the bias arising from confounding factors. Heterogeneity was assessed using I^2^ statistics with a threshold of 50% for at least moderate heterogeneity. An a priori subgroup analysis of different types of surgery was planned in our protocol. However, we only examined data on orthopedic surgery in this study because the number of studies on other types of surgery were too few to allow a meaningful synthesis of evidence. By contrast, a subgroup analysis based on different adjustment methods was made. Publication bias would have been visually evaluated via funnel plots if 10 or more studies reported an outcome. However, tests for funnel plot asymmetry were not performed because the number of included studies was <10 for each outcome.

## 3. Results

### 3.1. Literature Retrieval and Summary of Included Articles

**Figure 1:**
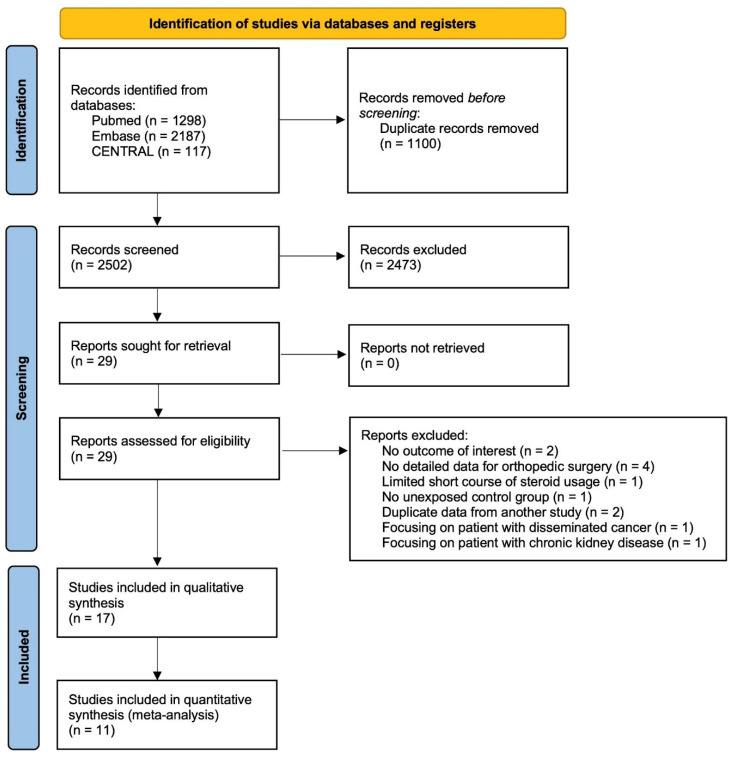
Preferred Reporting Items for Systematic Reviews and Meta-analyses (PRISMA) study flow diagram.

As seen in our illustration (Figure 1), 2502 articles were identified after removing duplicates. After scanning the title and abstracts, 29 studies remained for full-text assessment. The studies with a limited short course of steroid use and without outcomes of interest, detailed data for orthopedic surgery, or an unexposed control group were excluded. Two studies providing duplicated data from other studies, one study focusing on patients with disseminated cancer, and one study focusing on patients with chronic kidney diseases were also excluded. Finally, a total of 17 studies involving 1,546,562 patients who met our inclusion criteria were included.

The characteristics of the included studies are summarized in Table 1. These studies comprised patients derived from large representative databases in the United States and reported a variety of surgical outcomes. The risk of bias in each study was assessed according to NOS (Figure S1). Most studies were rated as having a low or unclear risk of bias. One study was assessed as having a high risk of bias in the “comparability of cohorts on the basis of the design or analysis” domain because no adjustment for confounding factors was made during the analysis.

### 3.2. Definition of Preoperative Chronic Steroid Use

Among the included studies, the patients classified as preoperative chronic steroid users mostly met the following criteria: patients required regular oral or parental corticosteroid medications for chronic conditions within 30 days prior to the index surgical procedures or being a surgical candidate [12,13,14,15,19,21,22,23,24,25,26,27,30,31]. Patients taking a limited short course (≤10 days) or taking topical, inhaled, and rectal steroids were excluded. Singla et al. [29] and Boylan et al. [20] used ICD-9 diagnosis code V5865 to identify patients with chronic steroid use preoperatively. In the study conducted by Roberts et al. [28], the definition for identifying the exposed cohort was not clearly stated.

### 3.3. Postoperative Outcomes of Interest

The outcomes of interest included mortality, overall complications, wound dehiscence, infectious complications, thromboembolism, re-admission, and re-operation. Most studies reported whether these outcomes occurred within 30 days after the index surgical procedures [12,13,14,15,19,21,22,23,24,25,26,27,30,31]. Some studies provided outcome data at different time points. In addition to 30-day outcomes, Boylan et al. [20] additionally provided 90-day outcomes for re-admission and thromboembolism events and 1- and 2-year outcomes for re-operation. Singla et al. [29] reported 90-day and 1-year outcomes for infectious complications and 1-year outcome for mortality. Roberts et al. [28] provided 3-month outcomes for wound dehiscence, sepsis, and surgical site infection, 6-month outcomes for re-operation and re-admission, and 1-year outcome for re-operation.

### 3.4. Primary Outcome: Mortality

**Figure 2:**
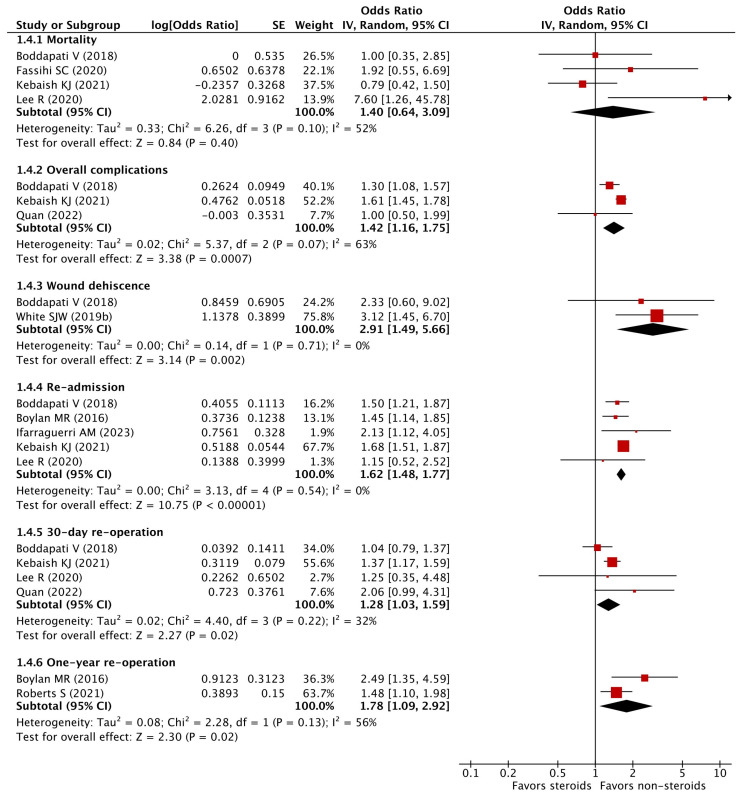
Adjusted odds ratios of 30-day mortality, overall complications, wound dehiscence, re-admission, and both 30-day and 1-year re-operation [12,15,19,20,22,25,27,28,31].

As shown in the supplemental table (Table S2), 11 studies reported crude ORs and 6 studies provided aORs for this outcome. After removing studies with possibly overlapping study subjects or reporting outcomes at different time points, four studies were included in our meta-analysis. The meta-analysis found no increase in 30-day mortality after receiving orthopedic surgery among patients with preoperative chronic steroid use (pooled aOR 1.40, 95% CI 0.64 to 3.09) (Figure 2). Moderate heterogeneity was detected across these studies (I^2^ = 52%).

### 3.5. Overall Complications

Seven studies reported crude ORs and five studies provided aORs for this outcome (see Table S2). Three studies remained for our meta-analysis after removing the studies with potentially overlapping data. The pooled analysis revealed increased odds of 30-day overall complications in patients with preoperative chronic steroid use after undergoing orthopedic surgery (pooled aOR 1.42, 95% CI 1.16 to 1.75) (Figure 2). Moderate heterogeneity was detected across these studies (I^2^ = 63%).

### 3.6. Wound Dehiscence

Of 12 studies investigating this outcome, the crude ORs and aORs were provided by 12 and 3 studies, respectively (see Table S2). Our meta-analysis on two studies revealed increased odds of 30-day wound dehiscence after receiving orthopedic surgery among patients with preoperative chronic steroid use (pooled aOR 2.91, 95% CI 1.49 to 5.66) without any statistical heterogeneity (I^2^ = 0%) (Figure 2).

### 3.7. Re-Admission

Ten studies provided crude ORs and six studies provided aORs for this outcome. The meta-analysis on five studies detected increased odds of 30-day re-admission among patients with preoperative chronic steroid use prior to orthopedic surgery (pooled aOR 1.62, 95% CI 1.48 to 1.77) (Figure 2). No statistical heterogeneity was detected across these studies (I^2^ = 0%).

### 3.8. Re-Operation

Nine studies evaluated this outcome within 30 days after surgery and two studies evaluated this outcome at different time points (6-, 12-, and 24-month) (see Table S2). For the outcome of 30-day re-operation, the meta-analysis on four studies with adjusted estimates showed increased odds of 30-day re-operation among patients with preoperative chronic steroid use (pooled OR 1.28, 95% CI 1.03–1.59) (Figure 2) after undergoing orthopedic surgery. Mild-to-moderate statistical heterogeneity was detected among these studies (I^2^ = 32%). For the outcome of 12-month re-operation, increased odds were also found in patients with preoperative chronic steroid use (pooled OR 1.78, 95% CI 1.09–2.92) with moderate statistical heterogeneity (I^2^ = 56%) (Figure 2).

### 3.9. Infectious Complications

**Figure 3:**
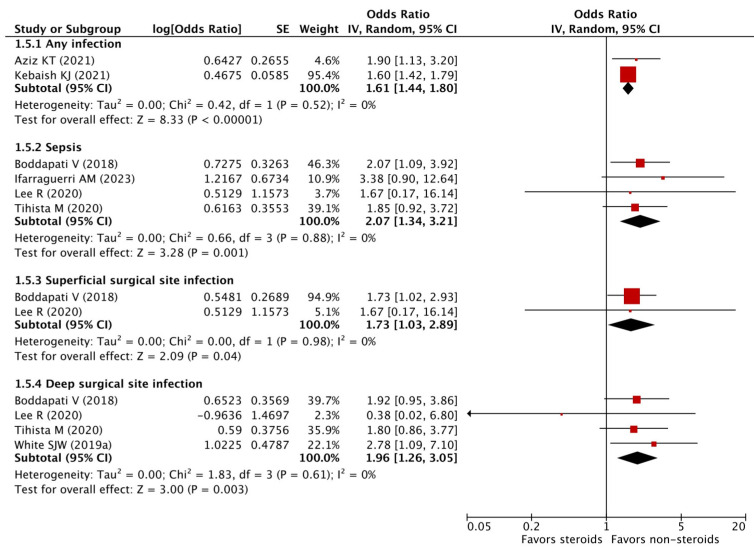
Adjusted odds ratios of infectious complications [12,13,14,19,22,25,30].

Fifteen studies investigated this outcome and reported either sepsis, superficial surgical site infection (SSI), deep SSI, and/or a composite outcome of sepsis, urinary tract infection, pneumonia, and SSI (Table S3). The meta-analysis on studies providing adjusted risk estimates showed that patients with preoperative chronic steroid use demonstrated increased odds of any infection (pooled OR 1.61, 95% CI 1.44–1.80), sepsis (pooled OR 2.07, 95% CI 1.34–3.21), superficial SSI (pooled OR 1.73, 95% CI 1.03–2.89), and deep SSI (pooled OR 1.96, 95% CI 1.26–3.05) after undergoing orthopedic surgery (Figure 3). No statistical heterogeneity was detected across these studies (I^2^ = 0%).

### 3.10. Thromboembolism

**Figure 4:**
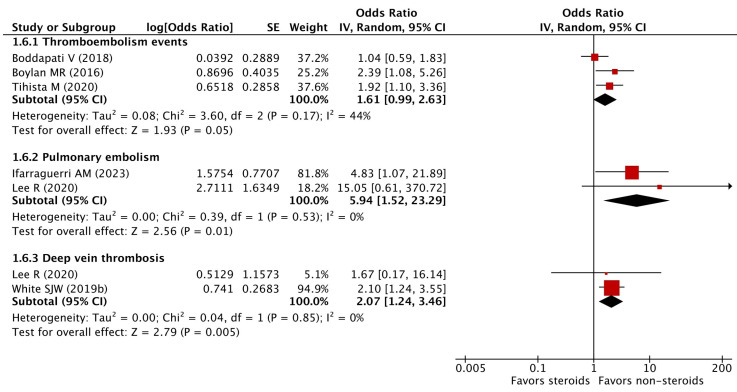
Adjusted odds ratios of thromboembolic events [14,19,20,22,25,31].

Thromboembolism events include pulmonary embolism, deep vein thrombosis, and their composition. Thirteen studies reported related outcomes (see Table S4) and the meta-analysis on adjusted estimates found no increase in 30-day thromboembolism events among patients with preoperative chronic steroid use prior to orthopedic surgery (pooled OR 1.61, 95% CI 0.99–2.63) with low statistical heterogeneity (I^2^ = 44%) (Figure 4). However, increased odds of 30-day pulmonary embolism (pooled OR 5.94, 95% CI 1.52–23.29) and deep vein thrombosis (pooled OR 2.07, 95% CI 1.24–3.46) were detected in patients with preoperative chronic steroid use (Figure 4).

### 3.11. Subgroup Analysis

The potential confounding factors were adjusted primarily by multivariate logistic regression or propensity score matching in our included studies (Table 1). Subgroup analyses were performed for each outcome based on the different adjustment methods. The effect sizes seemed to be greater in the multivariate regression group for re-admission, 30-day re-operation, sepsis, deep SSI, and thromboembolism events including both pulmonary embolism and deep vein thrombosis, but no significant differences were found between the different subgroups (see Table S5).

## 4. Discussion

To the best of our knowledge, this is the first systematic review and meta-analysis involving 1,546,562 subjects to assess the risk profile of postoperative outcomes in preoperative chronic steroid users undergoing orthopedic surgery. We found no increased risk of 30-day postoperative mortality following orthopedic surgery among patients with preoperative chronic steroid use. For secondary outcomes, an increased risk of 30-day overall complications, wound dehiscence, infectious complications, re-admission, and both 30-day and 1-year re-operation was detected. No significant increase in composite thromboembolic events was found in chronic steroid users. However, increased odds of 30-day pulmonary embolism and deep vein thrombosis were detected in chronic steroid users undergoing orthopedic surgery.

The impact of preoperative chronic steroid use on postoperative outcomes has been investigated and the link between preoperative chronic steroid use and worse surgical outcomes was established in some studies [9,10,11]. However, since the risk and outcomes of different types of surgical procedures are inherently different, the results could not be generalized to every surgical specialty. In this first meta-analysis focusing on orthopedic surgery, we included cohort studies but not case-control or cross-sectional studies because the temporal and causal relationship can only be clearly assessed in the former study design.

In the included studies, preoperative chronic steroid users are mostly defined as patients who required systemic steroids for chronic conditions within 30 days before the index surgical procedure with a duration of more than 10 days. A small number of studies simply capture the steroid users by the ICD code without a specific definition [20,29]. The adverse effects of steroids are both time- and dose-dependent. Long-term and higher doses of systemic steroids are believed to be more harmful than short-term and lower doses of steroids. However, the effect of hypophyseal pituitary adrenal axis suppression may develop as early as the first two weeks of steroid use and the patient may become immunosuppressed after using systemic steroids for more than one week [2]. In one cohort study, increased risk of sepsis, VTE, and fracture were detected among the patients who took systemic steroids even for <1 month [32]. Therefore, we only excluded studies with a limited course of preoperative steroid use because the side effects of steroids may occur as early as the first one to two weeks. However, we suppose that the association between preoperative steroid use and worse postoperative outcomes in orthopedic surgery may be stronger if the patients take preoperative steroids for a longer period.

In our analysis, studies reporting only crude ORs were excluded from our meta-analysis because the preoperative steroid users in these studies were usually more vulnerable with an older age or higher American Society for Anesthesiologists (ASA) classification, which might have biased the risk estimates. In addition, studies whose subjects were derived from the same database with the same surgical procedure and shorter study period as another study were also excluded from the meta-analysis to prevent the inclusion of overlapping subjects. Considering that different methods were applied for adjusting potential confounding factors, subgroup analysis based on different adjustment methods was conducted. However, there were limited studies in each subgroup and no significant differences were detected among them.

In this study, we demonstrated the exact risk profile of preoperative steroid use in patients undergoing orthopedic surgeries. In orthopedic surgery, pre-operative chronic steroid use was associated with poorer surgical outcomes, including overall complications, wound complications, infectious complications, and higher rates of re-operation, re-admission, and thromboembolic events. However, the exact pathogenesis for this association has not yet been conclusively determined and we provide some possible reasons as follows.

First, one possible explanation is the complex interplay in human stress response among long-term steroid administration, anesthetic procedures, and surgical intervention. Reactive oxygen species (ROS) play an important role in the normal cellular function of human bodies. The overproduction of ROS and the disability of antioxidant defense mechanisms to detoxify these reactive materials lead to a harmful state known as oxidative stress. The corticosteroids theoretically inhibit the production of ROS and display antioxidant properties. However, long-term administration of corticosteroids paradoxically increases the oxidative stress [33]. In a meta-analysis focusing on corticosteroid-induced physiological stress, the effect of increased oxidative stress is most prominent after three weeks of administration [34].

In addition, during surgery, local and systemic inflammatory responses are elicited with increased production of pro-inflammatory cytokines like interleukin (IL)-1, IL-2, IL-6, and IL-8, which also results in the increase in oxidative stress [35]. The increased oxidative stress during surgery is related to multiple organ dysfunction with higher risks of pulmonary, cardiac, infectious, hepatic, and nephritic complications [35]. Aside from the extent of the surgery, different types of induction anesthetic agents or different anesthetic procedures also have different effects on the oxidative stress [36,37]. For common inhaled anesthetics like sevoflurane and desflurane, increased oxidative stress was observed [37]. By contrast, among the intravenous anesthetics, propofol showed the greatest potential to mitigate inflammation and oxidative stress [37]. Considering different anesthetic procedures, higher pro-inflammatory cytokine production and greater oxidative stress were found in the general anesthesia group compared with the spinal anesthesia group for patients receiving orthopedic surgery in one study [36]. Therefore, the preoperative high oxidative stress status and the varying degrees of stress response during anesthesia and orthopedic surgery may both contribute to the higher rate of postoperative complications in patients with chronic steroid use.

Secondly, long-term systemic corticosteroid use inherently has huge impacts on different systems. For the immune system, cell-mediated immunity was impaired in long-term systemic steroid users and predisposed them to infection [38]. The monocyte function may also be affected, but it resolves rapidly after stopping medication [38]. These effects on the immune system are dose-related and patients with a daily dose of less than 10 mg (prednisone equivalent dose) or a cumulative dose of less than 700 mg may avoid these side effects [39]. For wound healing, corticosteroids have proven that they affect all major steps of the wound-healing process [5,6]. In animal models, an average 30% reduction in wound tensile strength was observed at cortisone doses of 15 to 40 mg/kg/day [40,41,42,43]. Furthermore, patients with Cushing’s syndrome, which involves chronic exposure to excessive adrenocorticoids, exhibited a 40% reduction in wound tensile strength compared with the healthy population [44,45]. Considering clinical studies, the results suggest that the wound complication rates may increase by 2- to 5-fold when preoperative corticosteroid treatment exceeds 30 days at a daily prednisone dose of 40 mg or greater [7]. In contrast, there is no clinical evidence to suggest that high-dose corticosteroid administration for less than 10 days adversely interferes with the wound-healing process [7]. Hence, it is not surprising that we observed poorer surgical outcomes, including infectious complications and wound dehiscence, in chronic steroid users.

Third, for VTE, whether the use of corticosteroids is a contributing factor is a challenging question, because the common medical conditions for prescribing corticosteroids are also frequently the risk factors associated with VTE. Several large population-based studies have indicated that the risk of VTE is significantly higher, with approximately a threefold increase, among users of steroids, especially during the initial period of use [32,46,47]. In the field of orthopedic surgery, venous thromboembolism is one of the most important complications. Identifying a modifiable predictive factor is essential for effective prophylaxis. Nonetheless, existing evidence does not strongly support a connection between chronic steroid use and an elevated risk of VTE in orthopedic surgery.

Last but not least, the connection between chronic steroid use and adverse surgical outcomes may be transitive. Prolonged corticosteroid administration is a recognized contributing factor to the development of osteoporosis, which in turn has been associated with increased bone fragility and poorer surgical outcomes [48]. Subgroup analysis based on osteoporosis status could potentially clarify the exact relationship between chronic steroid use, osteoporosis, and unfavorable surgical outcomes in orthopedic procedures. Unfortunately, we were unable to perform this analysis because of the absence of detailed data encoding osteoporosis conditions.

There are a few limitations in our study. First, as the included studies were exclusively from the United States, the generalizability of our results to other countries is uncertain. Second, the included studies did not provide information on the exact dose and duration of steroid use. Therefore, we were unable to perform a subgroup analysis to delineate the dose–response relationship. Third, mild-to-moderate heterogeneity was found in our analysis among some studies. However, we could not investigate the heterogeneity by conducting a subgroup analysis based on the patient’s surgical risk (e.g., ASA classification) as planned in our protocol because the included studies did not provide stratified data based on the patient’s surgical risk. Fourth, the OR and 95% CI on the ‘30-day mortality’ outcome provided by Fassihi et al. [15] seemed not to be accordant, but the author did not reply to our e-mail requesting the original data. Nevertheless, performing a sensitivity analysis by removing this study did not change the direction of our results. Fifth, though no increased odds of mortality and thromboembolic events were detected, wide CIs were demonstrated in these outcomes and the numbers of events were low. The imprecision in these outcomes leads to inconclusiveness based on current evidence. Therefore, more studies investigating these outcomes are warranted. Sixth, the role of chronic steroid use in osteoporosis and worse surgical outcomes cannot be delineated by subgroup analysis because of the absence of data in the included studies.

Our study provides valuable insights into the broad range of effects associated with pre-operative chronic steroid use on postoperative outcomes in orthopedic surgery. These findings underscore the significance of thorough risk assessment when planning surgery for patients on chronic steroids. For both anesthesiologists and orthopedic surgeons, the results of this study can serve as a benchmark in the clinical decision-making process, assisting them in evaluating the trade-off between benefits and potential risks for patients using chronic steroids. For patients under chronic steroid prescription, health care providers can inform them of the additional surgical risk revealed in this study, helping them to make informed decisions and actively participate in the management planning.

## 5. Conclusions

In conclusion, this study demonstrated the effects of preoperative chronic steroid use on postoperative outcomes in orthopedic surgery. Patients taking steroids preoperatively have an increased risk of worse surgical outcomes after undergoing orthopedic surgery. Multidisciplinary pre-operative patient counseling and risk stratification are crucial in this population. However, whether these effects become more prominent in high-risk patients is still unknown. Further dose–response studies should be conducted to elucidate this question.

## Figures and Tables

**Table 1:** Characteristics of the included studies.

Author (Year)	Database	Study Period	Expose/Control	Surgical Procedure	CPT or ICD Codes *	Outcome of Interest	Adjustment Method
Aziz KT et al. (2021) [13]	ACS-NSQIP	2011–2018	Steroids: 1662 Non-steroids: 98,308	Shoulder surgery (Exclude arthroplasty)	29827, 23410, 23412, 29828, 29430, 24342, 23020, 23130, 23415, 23420, 29826, 23420, 29824, 29805, 29806, 29807, 29825, 29822, 29823, 29819, 29820, 29821, 23455, 23460, 23465, 23662, 23466	Overall complications, infectious complications	Multivariable logistic regression
Boddapati V et al. (2018) [19]	ACS-NSQIP	2005–2015	Steroids: 3714 Non-steroids: 97,818	Total hip arthroplasty	27130	Mortality, overall complications, wound dehiscence, infectious complications, thromboembolism, re-admission, re-operation	Propensity score matching
Boylan MR et al. (2016) [20]	SPARCS	2003–2010	Steroids: 402 Non-steroids: 104,720	Primary total hip arthroplasty	Inclusion: ICD-9: 81.51, 00.74, 00.75, 00.76, 00.77 Exclusion: ICD-9: 899, 88 (exclude)	Thromboembolism, re-admission, re-operation	Propensity score matching
Cloney MB et al. (2018) [21]	ACS-NSQIP	2006–2013	Steroids: 353 Non-steroids: 8139	Posterior lumbar fusion	Inclusion: 22612 Exclusion: 22849, 22850, 22852, 22855; 0090T, 0092T, 0093T, 0095T, 0096T, 0098T, 0163T, 0164T, 0165T, 0163T, 0164T, 0165T, 0195T, 0196T, 0202T	Overall complications, wound dehiscence, infectious complications, thromboembolism, re-admission, re-operation	Multivariable logistic regression
Fassihi SC et al. (2020) [15]	ACS-NSQIP	2007–2016	Steroids: 474 Non-steroids: 10,499	Revision total knee arthroplasty ^†^	27486, 27487, 27488	Mortality, wound dehiscence, infectious complications, re-operation	Multivariable logistic regression
Ifarraguerri AM et al. (2023) [22]	ACS-NSQIP	2006–2018	Steroids: 178 Non-steroids: 10,153	ORIF for ankle fractures	CPT: 27766, 27792, 27814, 27822, 27823 ICD-9: 824.0–824.9 ICD-10: S82.5, S82.6, S82.84, S82.85, S82.87, S82.89	Mortality, wound dehiscence, infectious complications, thromboembolism, re-admission, re-operation	Multivariable logistic regression
Kantar RS et al. (2015) [23]	ACS-NSQIP	2008–2012	Steroids: 6575 Non-steroids: 223,029	All major surgical procedures stratified by subspecialty (orthopedic surgery)	Not specified	Thromboembolism	Multivariable logistic regression
Kebaish KJ et al. (2021) [12]	ACS-NSQIP	2005–2016	Steroids: 5243 Non-steroids: 135,276	Elective posterior lumbar spine surgery ^‡^	Inclusion: 63005, 63012, 63017, 63030, 63035, 63042, 63044, 63047, 63048; 22612, 22614; 22630, 22632, 22633, 22634 Exclusion: 22558, 22585, 22845, 22846, 22847, 22853	Mortality, overall complications, wound dehiscence, infectious complications, thromboembolism, re-admission, re-operation	Propensity score matching and Multivariable logistic regression
Kittle H et al. (2020) [24]	ACS-NSQIP	2010–2017	Steroids: 14,774 Non-steroids: 388,792	Total joint arthroplasty ^§^	27447, 27130, 27134, 27137, 27138, 27486, 27487	Mortality, wound dehiscence, infectious complications, thromboembolism, re-admission	NA
Lee R et al. (2020) [25]	ACS-NSQIP	2005–2016	Steroids: 198 Non-steroids: 5179	Anterior cervical discectomy and fusion	22551, 22554	Mortality, wound dehiscence, infectious complications, thromboembolism, re-admission, re-operation	Propensity score matching
Newton WN et al. (2023) [26]	ACS-NSQIP	2005–2020	Steroids: 93 Non-steroids: 1205	Salvage operations for wrist arthritis ^‖^	25215, 25820, 25825, 25800, 25805, 25810, 25446	Overall complications, wound dehiscence, infectious complications, thromboembolism, re-admission, re-operation	Multivariable logistic regression
Quan et al. (2022) [27]	ACS-NSQIP	2007–2018	Steroids: 360 Non-steroids: 16,145	ORIF for DRFs	25607, 25608, 25609	Mortality, overall complications, infectious complications, thromboembolism, re-operation	Multivariable logistic regression
Roberts S et al. (2021) [28]	PearlDiver	2007–2017	Steroids (>6 m) ^¶^: 2611 Steroids (<6 m): 2800 Non-steroids: 3704	Posterior/transforaminal lumbar interbody fusion	22630, 22632, 22633, 22634	Wound dehiscence, infectious complications, re-admission, re-operation	Matched unexposed cohort
Singla A et al. (2019) [29]	PearlDiver	2005–2012	Steroids: 11,687 Non-steroids: 348,318	Elective posterior lumbar fusion	ICD-9- P-8106, ICD-9-P-8107, ICD-9-P-8108	Mortality, infectious complications	Multivariable logistic regression
Tihista M et al. (2020) [14]	ACS-NSQIP	2005–2016	Steroids: 1044 Non-steroids: 25,690	Lumbar decompression procedures	63005, 63017, 63030, 63042, 63047	Mortality, overall complications, wound dehiscence, infectious complications, thromboembolism, re-admission, re-operation	Multivariable logistic regression
White SJW et al. (2019a) [30]	ACS-NSQIP	2008–2015	Steroids: 289 Non-steroids: 9194	Elective anterior lumbar fusion	22558	Mortality, wound dehiscence, infectious complications, thromboembolism	Multivariable logistic regression
White SJW et al. (2019b) [31]	ACS-NSQIP	2008–2015	Steroids: 418 Non-steroids: 7518	Elective adult spinal deformity surgery	22595, 22600, 22612, 22630, 22633, 22551, 22554, 22558	Mortality, wound dehiscence, infectious complications, thromboembolism	Multivariable logistic regression

* Use CPT code primarily if no other descriptions. ^†^ Patients who had an infectious etiology for revision were excluded from this study. ^‡^ Patients undergoing surgery emergently or because of neoplasm, trauma, or infection were excluded. Posterior lumbar spine surgery included decompression and/or fusion. ^§^ Including primary and revision total knee arthroplasty and primary and revision total hip arthroplasty. ^‖^ The operation methods included proximal row carpectomy, four-corner fusion, total wrist arthroplasty, total wrist arthrodesis. ^¶^ This study divided the steroid-using group into two groups with exposure >6 months or <6 months (exposure < 6 months were presented first). CPT, Current Procedural Terminology; ICD, International Classification of Disease; ACS-NSQIP, American College of Surgeons–National Surgical Quality Improvement Program; SPARCS, The New York Statewide Planning and Research Cooperative System; ORIF, open reduction and internal fixation; DRFs, distal radius fractures.

## Data Availability

The datasets generated and/or analyzed during the current study are available from the corresponding author upon reasonable request.

## Supplementary Materials

The following supporting information can be downloaded at: https://www.mdpi.com/article/10.3390/ph16091328/s1, Figure S1: Risk of bias of included cohort studies; Table S1: Detailed search strategy; Table S2: Summary of crude and adjusted risk estimates of mortality, overall complications, wound dehiscence, re-admission, and re-operation; Table S3: Summary of crude and adjusted risk estimates of infectious complications; Table S4: Summary of crude and adjusted risk estimates of thromboembolism events; Table S5: Subgroup analysis for each outcome based on adjustment methods of confounding factors.
