# Supplementary material for: Effects of Preoperative Chronic Steroid Use on Postoperative Outcomes in Orthopedic Surgery: A Systematic Review and Meta-Analysis
Source: Pharmaceuticals (Basel). 2023 Sep 20;16(9):1328. doi: 10.3390/ph16091328 (PMC10536822; doi:10.3390/ph16091328)
Supplement: Supplementary file 1 [file pharmaceuticals-16-01328-s001.zip › pharmaceuticals-2585466-supplementary.pdf]

Supplementary materials

Table S1. Detailed Search Strategy

Pubmed & CENTRAL search strategy

1. "chronic steroid*"[Title/Abstract] OR "chronic corticosteroid*"[Title/Abstract] OR "chronic predniso*"[Title/Abstract] OR "long term steroid*"[Title/Abstract] OR "long-term steroid*"[Title/Abstract] OR "pre-operative steroid*"[Title/Abstract] OR "preoperative steroid*"[Title/Abstract] OR "long term corticosteroid*"[Title/Abstract] OR "long-term corticosteroid*"[Title/Abstract] OR "pre-operative corticosteroid*"[Title/Abstract] OR "preoperative corticosteroid*"[Title/Abstract] OR "long term predniso*"[Title/Abstract] OR "long-term predniso*"[Title/Abstract] OR "preoperative predniso*"[Title/Abstract] OR "pre-operative predniso*"[Title/Abstract]
2. "surgical procedures, operative"[MeSH Terms] OR "surgery"[Text Word] OR "operation"[Text Word]
3. 1 AND 2

Embase search strategy

1. 'chronic steroid*'
2. 'chronic corticosteroid*'
3. 'chronic predniso*'
4. 'long-term steroid*'
5. 'long term steroid*'
6. 'pre-operative steroid*'
7. 'preoperative steroid*'
8. 'long-term corticosteroid*'
9. 'long term corticosteroid*'
10. 'pre-operative corticosteroid*'
11. 'preoperative corticosteroid*'
12. 'long-term predniso*'
13. 'long term predniso*'
14. 'pre-operative predniso*'
15. 'preoperative predniso*'
16. #1 OR #2 OR #3 OR #4 OR #5 OR #6 OR #7 OR #8 OR #9 OR #10 OR #11 OR #12 OR #13 OR #14 OR #15
17. Operation
18. Surgery
19. #17 OR #18
20. #16 AND #19

Figure S1. Risk of bias of included cohort studies. Green dots indicate low risk of bias, yellow dots for unclear risk of bias and red dots for high risk of bias.

	Representativeness of the exposed cohort	Selection of the non exposed cohort	Ascertainment of exposure	Demonstration that outcome of interest was not present at start of study	Comparability of cohorts on the basis of the design or analysis	Assessment of outcome	Was follow-up long enough for outcomes to occur	Adequacy of follow up of cohorts
Aziz KT (2021)	+	+	+	+	?	+	+	+
Boddapati V (2018)	+	+	+	+	+	+	+	+
Boylan MR (2016)	+	+	?	+	+	?	+	+
Cloney MB (2018)	+	+	+	+	?	+	+	+
Fassihi SC (2020)	+	+	+	+	?	+	+	+
Ifarraguerri AM (2023)	+	+	+	+	?	+	+	+
Kantar RS (2015)	+	+	+	+	+	+	+	+
Kebaish KJ (2021)	+	+	+	+	+	+	+	+
Kittle H (2020)	+	+	+	+	−	+	+	+
Lee R (2020)	+	+	?	+	+	?	+	+
Newton WN (2023)	+	+	+	+	?	+	+	+
Quan (2022)	+	+	+	+	?	+	+	+
Roberts S (2021)	+	+	?	+	+	?	+	+
Singla A (2019)	?	+	?	+	+	?	+	+
Tihista M (2020)	+	+	+	+	?	+	+	+
White SJW (2019a)	+	+	+	+	?	+	+	+
White SJW (2019b)	+	+	+	+	?	+	+	+

Table S2. Summary of Crude and Adjusted Risk Estimates of Mortality, Overall Complications, Wound Dehiscence, Re-Admission, and Re-Operation.

Study	Outcome of interest	Event/total (n/N)	Crude OR [95%CI]	Adjusted OR [95%CI]	Adjusted factors
Mortality					
Boddapati V, et al (2018)	30-day mortality	Steroid group: 9/3714<br>Non-steroid group: 166/97818	1.43 [0.73, 2.80]	1.00 [0.35, 2.85]	Age, sex, BMI, comorbidities, ASA class, anesthesia type, operative duration (Matching)
Fassihi SC, et al (2020)	30-day mortality	Steroid group: 3/474<br>Non-steroid group: 20/10499	3.34 [0.99, 11.27]	1.92 [0.55, 6.69]¶	Not well documented
Ifarraguerri AM, et al (2023)	30-day mortality	Steroid group: 0/178<br>Non-steroid group: 25/10153	1.11 [0.07, 18.35]	NA	NA
Kebaish KJ, et al (2021)	30-day mortality	Steroid group: 10/5243<br>Non-steroid group: 200/135276	1.29 [0.68, 2.44]	0.79 [0.42, 1.50]	Age, sex, BMI, functional status, procedure type, and ASA class
Kittle H, et al (2020)	30-day mortality	Steroid group: 0/14774<br>Non-steroid group: 6/388792	2.02 [0.11, 35.93]	NA	NA
Lee R, et al (2020)	30-day mortality	Steroid group: 3/198<br>Non-steroid group: 10/5179	7.95 [2.17, 29.12]	7.60 [1.26, 45.78]	Age, sex, smoking, comorbidities, ASA class (Matching)
Quan, et al (2022)	30-day mortality	Steroid group: 1/360<br>Non-steroid group: 17/16145	2.64 [0.35, 19.91]	NA	NA
Singla A, et al (2019)	1-year mortality	Steroid group: 39/11687<br>Non-steroid group: 326/348318	3.57 [2.56, 4.98]	2.06 [1.13, 3.78]	Comorbidities
Tihista M, et al (2020)	30-day mortality	Steroid group: 1/1044<br>Non-steroid group: 34/25690	0.72 [0.10, 5.29]	NA	NA
White SJW, et al (2019a)	30-day mortality	Steroid group: 1/289<br>Non-steroid group: 21/9194	1.52 [0.20, 11.31]	NA	NA

White SJW, et al (2019b)	30-day mortality	Steroid group: 14/418<br>Non-steroid group: 59/7518	4.38 [2.43, 7.91]	2.42 [1.30, 4.51]	Age, sex, comorbidities, functional status, ASA class
Overall complications					
Aziz KT, et al (2021)	30-day complications	Steroid group: 57/1662<br>Non-steroid group: 885/98308	3.91 [2.98, 5.13]	NA	NA
Boddapati V, et al (2018)	30-day complications	Steroid group: 293/3714<br>Non-steroid group: 4764/97818	1.67 [1.48, 1.89]	1.30 [1.08, 1.57]	Age, sex, BMI, comorbidities, ASA class, anesthesia type, operative duration (Matching)
Cloney MB, et al (2018)	30-day complications	Steroid group: 51/353<br>Non-steroid group: 782/8139	1.59 [1.17, 2.16]	1.38 [1.01, 1.90]	Baseline demographics and clinical data
Kebaish KJ, et al (2021)	30-day complications	Steroid group: 466/5243<br>Non-steroid group: 6098/135276	2.07 [1.87, 2.28]	1.61 [1.45, 1.78]	Age, sex, BMI, functional status, procedure type, and ASA class
Newton WN, et al (2023)	30-day complications	Steroid group: 5/93<br>Non-steroid group: 14/1205	4.83 [1.70, 13.73]	NA	NA
Quan, et al (2022)	30-day complications	Steroid group: 10/360<br>Non-steroid group: 198/16145	2.30 [1.21, 4.38]	0.997 [0.499, 1.992]	Age, sex, BMI, functional status, ASA class, comorbidities
Tihista M, et al (2020)	30-day complications	Steroid group: 96/1044<br>Non-steroid group: 1258/25690	1.97 [1.58, 2.44]	1.54 [1.23, 1.94]	Age, sex, BMI, functional status, ASA class, and comorbidities
Wound dehiscence					

Boddapati V, et al (2018)	30-day wound dehiscence	Steroid group: 8/3714<br>Non-steroid group: 108/97818	1.95 [0.95, 4.01]	2.33 [0.60, 9.02]	Age, sex, BMI, comorbidities, ASA class, anesthesia type, operative duration (Matching)
Cloney MB, et al (2018)	30-day wound dehiscence	Steroid group: 1/353<br>Non-steroid group: 29/8139	0.79 [0.11, 5.85]	NA	NA
Fassihi SC, et al (2020)	30-day wound dehiscence	Steroid group: 2/474<br>Non-steroid group: 42/10499	1.05 [0.25, 4.37]	NA	NA
Ifarraguerri AM, et al (2023)	30-day wound dehiscence	Steroid group: 1/178<br>Non-steroid group: 35/10153	1.63 [0.22, 11.99]	NA	NA
Kebaish KJ, et al (2021)	30-day wound dehiscence	Steroid group: 19/5243<br>Non-steroid group: 299/135276	1.64 [1.03, 2.61]	NA	NA
Kittle H, et al (2020)	30-day wound dehiscence	Steroid group: 56/14774<br>Non-steroid group: 668/388792	2.21 [1.68, 2.91]	NA	NA
Lee R, et al (2020)	30-day wound dehiscence	Steroid group: 0/198<br>Non-steroid group: 2/5179	5.22 [0.25, 109.02]	NA	NA
Newton WN, et al (2023)	30-day wound dehiscence	Steroid group: 0/93<br>Non-steroid group: 1/1205	4.29 [0.17, 106.14]	NA	NA
Roberts S, et al (2021)	3-month wound dehiscence	Steroid group (< 6m)[a]: 32/2800<br>Steroid group (> 6m): 28/2611<br>Non-steroid group: 39/3704	1.09 [0.68, 1.74][b]<br>1.02 [0.63, 1.66][c]<br>1.07 [0.64, 1.78][d]	0.96 [0.55, 1.69][b]<br>0.88 [0.49, 1.58][c]<br>1.09 [0.61, 1.95][d]	Age, sex, and Charlson Comorbidity Index (Matching)
Tihista M, et al (2020)	30-day wound dehiscence	Steroid group: 2/1044<br>Non-steroid group: 73/25690	0.67 [0.17, 2.75]	NA	NA
White SJW, et al (2019a)	30-day wound dehiscence	Steroid group: 2/289<br>Non-steroid group: 22/9194	2.91 [0.68, 12.41]	NA	NA

White SJW, et al (2019b)	30-day wound dehiscence	Steroid group: 8/418<br>Non-steroid group: 44/7518	3.31 [1.55, 7.09]	3.12 [1.45, 6.70]	Age, sex, comorbidities, functional status, ASA class
Re-admission					
Boddapati V, et al (2018)	30-day readmission	Steroid group: 242/3714<br>Non-steroid group: 3453/97818	1.90 [1.66, 2.18]	1.50 [1.21, 1.87]	Age, sex, BMI, comorbidities, ASA class, anesthesia type, operative duration (Matching)
Boylan MR, et al (2016)	30-day readmission	Steroid group: NA/402<br>Non-steroid group: NA/104720	NA	1.45 [1.14, 1.85]	Age, sex, race, year of surgery (Matching)
	90-day readmission	Steroid group: NA/402<br>Non-steroid group: NA/104720	NA	1.37 [1.09, 1.73]	Age, sex, race, year of surgery (Matching)
Cloney MB, et al (2018)	30-day readmission	Steroid group: 33/353<br>Non-steroid group: 488/8139	1.62 [1.12, 2.34]	NA	NA
Ifarraguerri AM, et al (2023)	30-day readmission	Steroid group: 13/178<br>Non-steroid group: 197/10153	3.98 [2.23, 7.13]	2.13 [1.12, 4.05]	Age, sex, BMI, comorbidities, ASA class
Kebaish KJ, et al (2021)	30-day readmission	Steroid group: 414/5243<br>Non-steroid group: 5389/135276	2.07 [1.86, 2.29]	1.68 [1.51, 1.87]	Age, sex, BMI, functional status, procedure type, and ASA
Kittle H, et al (2020)	30-day readmission	Steroid group: 50/14774<br>Non-steroid group: 844/388792	1.56 [1.17, 2.08]	NA	NA
Lee R, et al (2020)	30-day readmission	Steroid group: 8/198<br>Non-steroid group: 177/5179	1.19 [0.58, 2.45]	1.15 [0.52, 2.52]	Age, sex, smoking, comorbidities, ASA class (PS-matching)
Newton WN, et al (2023)	30-day readmission	Steroid group: 2/93<br>Non-steroid group: 20/1205	1.30 [0.30, 5.66]	NA	NA

Roberts S, et al (2021)	6-month readmission	Steroid group (< 6m)[a]: 499/2800<br>Steroid group (> 6m): 433/2611<br>Non-steroid group: 550/3704	1.24 [1.09, 1.42][b]<br>1.14 [0.99, 1.31][c]<br>0.82 [0.42, 1.61][d]	1.14 [0.97, 1.34][b]<br>1.00 [0.85, 1.17][c]<br>1.14 [0.98, 1.34][d]	Age, sex, and Charlson Comorbidity Index (Matching)
Tihista M, et al (2020)	30-day readmission	Steroid group: 11/1044<br>Non-steroid group: 126/25690	2.16 [1.16, 4.01]	NA	NA
Re-operation					
Boddapati V, et al (2018)	Reoperation within 30 days	Steroid group: 115/3714<br>Non-steroid group: 1966/97818	1.56 [1.29, 1.89]	1.04 [0.79, 1.37]	Age, sex, BMI, comorbidities, ASA class, anesthesia type, operative duration (Matching)
Boylan MR, et al (2016)	Revision within 12-month	Steroid group: NA/402<br>Non-steroid group: NA/104720	NA	2.49 [1.35, 4.59]	Age, sex, race, year of surgery (Matching)
	Revision within 24-month	Steroid group: NA/402<br>Non-steroid group: NA/104720	NA	2.04 [1.19, 3.50]	Age, sex, race, year of surgery (Matching)
Cloney MB, et al (2018)	Reoperation within 30 days	Steroid group: 23/353<br>Non-steroid group: 292/8139	1.87 [1.21, 2.90]	NA	NA
Fassihi SC, et al (2020)	Reoperation within 30 days	Steroid group: 14/474<br>Non-steroid group: 332/10499	0.93 [0.54, 1.60]	NA	NA
Ifarraguerri AM, et al (2023)	Reoperation within 30 days	Steroid group: 4/178<br>Non-steroid group: 160/10153	1.44 [0.53, 3.92]	NA	NA
Kebaish KJ, et al (2021)	Reoperation within 30 days	Steroid group: 188/5243<br>Non-steroid group: 3098/135276	1.59 [1.37, 1.84]	1.37 [1.17, 1.59]	Age, sex, BMI, functional status, procedure type, and ASA class
Lee R, et al (2020)	Reoperation within 30 days	Steroid group: 3/198<br>Non-steroid group: 81/5179	0.97 [0.30, 3.09]	1.25 [0.35, 4.48]	Age, sex, smoking, comorbidities, ASA class (Matching)

Newton WN, et al (2023)	Reoperation within 30 days	Steroid group: 1/93<br>Non-steroid group: 8/1205	1.63 [0.20, 13.14]	NA	NA
Quan, et al (2022)	Reoperation within 30 days	Steroid group: 8/360<br>Non-steroid group: 164/16145	2.21 [1.08, 4.54]	2.06 [0.99, 4.31]	Age, sex, BMI, functional status, ASA class, comorbidities
Roberts S, et al (2021)	Revision within 6-month	Steroid group (< 6m)[a]: 109/2800<br>Steroid group (> 6m): 71/2611<br>Non-steroid group: 103/3704	1.42 [1.08, 1.86][b]<br>0.98 [0.72, 1.33][c]<br>1.45 [1.07, 1.96][d]	1.33 [0.95, 1.86][b]<br>0.95 [0.66, 1.36][c]<br>1.40 [1.00, 1.96][d]	Age, sex, and Charlson Comorbidity Index (Matching)
	Revision within 12-month	Steroid group (< 6m)[a]: 150/2800<br>Steroid group (> 6m): 99/2611<br>Non-steroid group: 136/3704	1.49 [1.17, 1.88][b]<br>1.03 [0.79, 1.35][c]<br>1.44 [1.11, 1.86][d]	1.48 [1.10, 1.98][b]<br>1.08 [0.79, 1.47][c]<br>1.37 [1.03, 1.82][d]	Age, sex, and Charlson Comorbidity Index (Matching)
Tihista M, et al (2020)	Reoperation within 30 days	Steroid group: 0/1044<br>Non-steroid group: 9/25690	1.29 [0.08, 22.25]	NA	NA

¶CIs were calculated based on the provided odds ratio and its standard error, which was deviated from the reported data in original articles.

[a]This study divided steroids using group into two groups with exposure > 6 months or < 6 months (exposure < 6 months were presented first)

[b]Exposure < 6 months compared with non-exposure group

[c]Exposure > 6 months compared with non-exposure group

[d]Exposure < 6 months compared with exposure > 6 months

OR, odds ratio; BMI, body mass index; ASA class, American Society of Anesthesiologists Classification

Table S3. Summary of Crude and Adjusted Risk Estimates of Infectious Complications.

Study	Outcome of interest	Event/total (n/N)	Crude OR [95%CI]	Adjusted OR [95%CI]	Adjusted factors
Any infectious complications					
Aziz KT, et al (2021)	30-day sepsis, superficial and deep SSI	Steroid group: 17/1662 Non-steroid group: 393/98308	2.57 [1.58, 4.19]	1.9 [1.13, 3.20]	Demographics and comorbidities
Cloney MB, et al (2018)	30-day sepsis, UTI, pneumonia, superficial and deep SSI	Steroid group: 34/353 Non-steroid group: 414/8139	1.99 [1.38, 2.87]	1.65 [1.13, 2.41]	Baseline demographics and clinical data
Kebaish KJ, et al (2021)	30-day sepsis, UTI, pneumonia, superficial and deep SSI	Steroid group: 381/5243 Non-steroid group: 4953/135276	2.06 [1.85, 2.30]	1.60 [1.42, 1.79]	Age, sex, BMI, functional status, procedure type, and ASA class
Sepsis					
Boddapati V, et al (2018)	30-day sepsis	Steroid group: 31/3714 Non-steroid group: 274/97818	3.00 [2.06, 4.35]	2.07 [1.09, 3.92]	Age, sex, BMI, comorbidities, ASA class, anesthesia type, operative duration (Matching)
Cloney MB, et al (2018)	30-day sepsis	Steroid group: 9/353 Non-steroid group: 84/8139	2.51 [1.25, 5.03]	NA	NA
Fassihi SC, et al (2020)	30-day sepsis	Steroid group: 4/474 Non-steroid group: 39/10499	2.28 [0.81, 6.41]	NA	NA
Ifarraguerri AM, et al (2023)	30-day sepsis	Steroid group: 3/178 Non-steroid group: 28/10153	6.20 [1.87, 20.58]	3.38 [0.90, 12.64]	Age, sex, BMI, comorbidities, ASA class
Kebaish KJ, et al (2021)	30-day sepsis	Steroid group: 77/5243 Non-steroid group: 886/135276	2.26 [1.79, 2.86]	NA	NA
Kittle H, et al (2020)	30-day sepsis	Steroid group: 0/14774 Non-steroid group: 11/388792	1.14 [0.07, 19.42]	NA	NA

Lee R, et al (2020)	30-day sepsis	Steroid group: 1/198<br>Non-steroid group: 11/5179	2.38 [0.31, 18.56]	1.67 [0.17, 16.14]	Age, sex, smoking, comorbidities, ASA class (Matching)
Newton WN, et al (2023)	30-day sepsis	Steroid group: 0/93<br>Non-steroid group: 2/1205	2.57 [0.12, 54.01]	NA	NA
Quan, et al (2022)	30-day sepsis	Steroid group: 1/360<br>Non-steroid group: 13/16145	3.46 [0.45, 26.49]	NA	NA
Roberts S, et al (2021)	3-month sepsis	Steroid group (< 6m)[a]: 171/2800<br>Steroid group (> 6m): 116/2611<br>Non-steroid group: 177/3704	1.30 [1.04, 1.61][b]<br>0.93 [0.73, 1.18][c]<br>1.40 [1.10, 1.78][d]	1.16 [0.90, 1.51][b]<br>0.75 [0.56, 1.01][c]<br>1.54 [1.16, 2.05][d]	Age, sex, and Charlson Comorbidity Index (Matching)
Tihista M, et al (2020)	30-day sepsis	Steroid group: 10/1044<br>Non-steroid group: 103/25690	2.40 [1.25, 4.61]	1.85 [0.92, 3.72]	Age, sex, BMI, functional status, ASA class, and comorbidities
White SJW, et al (2019a)	30-day sepsis	Steroid group: 5/289<br>Non-steroid group: 50/9194	3.22 [1.27, 8.13]	NA	NA
White SJW, et al (2019b)	30-day sepsis	Steroid group: 10/418<br>Non-steroid group: 135/7518	1.34 [0.70, 2.57]	NA	NA
Surgical site infection (SSI)					
Boddapati V, et al (2018)	30-day superficial SSI	Steroid group: 38/3714<br>Non-steroid group: 665/97818	1.51 [1.09, 2.10]	1.73 [1.02, 2.93]	Age, sex, BMI, comorbidities, ASA class, anesthesia type, operative duration (Matching)
	30-day deep SSI	Steroid group: 25/3714<br>Non-steroid group: 284/97818	2.33 [1.54, 3.51]	1.92 [0.95, 3.86]	Age, sex, BMI, comorbidities, ASA class, anesthesia type, operative duration (Matching)
Cloney MB, et al (2018)	30-day superficial SSI	Steroid group: 7/353<br>Non-steroid group: 117/8139	1.39 [0.64, 3.00]	NA	NA
	30-day deep SSI	Steroid group: 6/353<br>Non-steroid group: 63/8139	2.22 [0.95, 5.16]	NA	NA

Fassihi SC, et al (2020)	30-day superficial SSI	Steroid group: 1/474<br>Non-steroid group: 71/10499	0.31 [0.04, 2.24]	NA	NA
	30-day deep SSI	Steroid group: 4/474<br>Non-steroid group: 53/10499	1.68 [0.60, 4.65]	NA	NA
Ifarraguerri AM, et al (2023)	30-day superficial SSI	Steroid group: 2/178<br>Non-steroid group: 70/10153	1.64 [0.40, 6.73]	NA	NA
	30-day deep SSI	Steroid group: 1/178<br>Non-steroid group: 33/10153	1.73 [0.24, 12.74]	NA	NA
Kebaish KJ, et al (2021)	30-day superficial SSI	Steroid group: 78/5243<br>Non-steroid group: 1225/135276	1.65 [1.31, 2.08]	NA	NA
	30-day deep SSI	Steroid group: 80/5243<br>Non-steroid group: 833/135276	2.50 [1.98, 3.15]	NA	NA
Kittle H, et al (2020)	30-day deep SSI	Steroid group: 54/14774<br>Non-steroid group: 727/388792	1.96 [1.48, 2.58]	NA	NA
	30-day SSI	Steroid group: 116/14774<br>Non-steroid group: 2126/388792	1.44 [1.19, 1.74]	NA	NA
	30-day any wound infection	Steroid group: 269/14774<br>Non-steroid group: 4247/388792	1.68 [1.48, 1.90]	NA	NA
Lee R, et al (2020)	30-day superficial SSI	Steroid group: 1/198<br>Non-steroid group: 15/5179	1.75 [0.23, 13.30]	1.67 [0.17, 16.14]	Age, sex, smoking, comorbidities, ASA class (Matching)
	30-day deep SSI	Steroid group: 0/198<br>Non-steroid group: 13/5179	0.96 [0.06, 16.27]	0.38 [0.02, 6.80]	Age, sex, smoking, comorbidities, ASA class (Matching)

Newton WN, et al (2023)	30-day SSI	Steroid group: 2/93<br>Non-steroid group: 8/1205	3.29 [0.69, 15.71]	NA	NA
Roberts S, et al (2021)	3-month SSI	Steroid group (< 6m)[a]: 76/2800<br>Steroid group (> 6m): 61/2611<br>Non-steroid group: 95/3704	1.06 [0.78, 1.44][b]<br>0.91 [0.66, 1.26][c]<br>1.17 [0.83, 1.64][d]	0.86 [0.59, 1.26][b]<br>0.74 [0.50, 1.10][c]<br>1.16 [0.77, 1.75][d]	Age, sex, and Charlson Comorbidity Index (Matching)
Singla A, et al (2019)	3-month SSI	Steroid group: 284/11687<br>Non-steroid group: 5725/348318	1.47 [1.31, 1.66]	1.74 [1.33, 1.92]	Comorbidities
	1-year SSI	Steroid group: 330/11687<br>Non-steroid group: 6682/348318	1.49 [1.33, 1.66]	1.88 [1.41, 2.01]	Comorbidities
Tihista M, et al (2020)	30-day superficial SSI	Steroid group: 15/1044<br>Non-steroid group: 225/25690	1.65 [0.97, 2.79]	NA	NA
	30-day deep SSI	Steroid group: 10/1044<br>Non-steroid group: 112/25690	2.21 [1.15, 4.23]	1.80 [0.86, 3.77]¶	Age, sex, BMI, functional status, ASA class, and comorbidities
White SJW, et al (2019a)	30-day superficial SSI	Steroid group: 3/289<br>Non-steroid group: 103/9194	0.93 [0.29, 2.94]	NA	NA
	30-day deep SSI	Steroid group: 5/289<br>Non-steroid group: 47/9194	3.43 [1.35, 8.68]	2.78 [1.09, 7.10]	Age, sex, comorbidities, functional status, ASA class, operative variables
White SJW, et al (2019b)	30-day superficial SSI	Steroid group: 4/418<br>Non-steroid group: 69/7518	1.04 [0.38, 2.87]	NA	NA
	30-day deep SSI	Steroid group: 6/418<br>Non-steroid group: 72/7518	1.51 [0.65, 3.48]	NA	NA

¶CIs were calculated based on the provided odds ratio and its standard error, which was deviated from the reported data in original articles.

[a]This study divided steroids using group into two groups with exposure > 6 months or < 6 months (exposure < 6 months were presented first)

[b]Exposure < 6 months compared with non-exposure group

[c]Exposure > 6 months compared with non-exposure group

[d]Exposure < 6 months compared with exposure > 6 months

OR, odds ratio; BMI, body mass index; ASA class, American Society of Anesthesiologists Classification; SSI, surgical site infection; UTI, urinary tract infection

Table S4. Summary of Crude and Adjusted Risk Estimates of Thromboembolism Events.

Study	Outcome of interest	Event/total (n/N)	Crude OR [95%CI]	Adjusted OR [95%CI]	Adjusted factors
Composite thromboembolism events					
Boddapati V, et al (2018)	DVT and PE within 30 days	Steroid group: 33/3714<br>Non-steroid group: 616/97818	1.41 [1.00, 2.01]	1.04 [0.59, 1.83]	Age, sex, BMI, comorbidities, ASA class, anesthesia type, operative duration (Matching)
Boylan MR, et al (2016)	DVT and PE within 30 days	Steroid group: NA/402<br>Non-steroid group: NA/104720	NA	2.39 [1.08, 5.26]	Age, sex, race, year of surgery (Matching)
	DVT and PE within 90 days	Steroid group: NA/402<br>Non-steroid group: NA/104720	NA	1.91 [1.03, 3.53]	Age, sex, race, year of surgery (Matching)
Cloney MB, et al (2018)	DVT and PE within 30 days	Steroid group: 7/353<br>Non-steroid group: 115/8139	1.41 [0.65, 3.05]	NA	NA
Kantar RS, et al	DVT and PE within 30 days	Steroid group: 107/6575<br>Non-steroid group: 2008/223029	1.82 [1.49, 2.21]	1.36 [1.11, 1.66]	Age, sex, race, BMI, comorbidities, ASA class, type of operation, etc.
Kebaish KJ, et al (2021)	DVT and PE within 30 days	Steroid group: 92/5243<br>Non-steroid group: 1016/135276	2.36 [1.90, 2.93]	NA	NA
Kittle H, et al (2020)	DVT and PE within 30 days	Steroid group: 57/14774<br>Non-steroid group: 1672/388792	0.90 [0.69, 1.17]	NA	NA
Quan, et al (2022)	DVT and PE within 30 days	Steroid group: 1/360<br>Non-steroid group: 11/16145	4.09 [0.53, 31.73]	NA	NA
Tihista M, et al (2020)	DVT and PE within 30 days	Steroid group: 14/1044<br>Non-steroid group: 154/25690	2.25 [1.30, 3.91]	1.92 [1.20, 3.36]	Age, sex, BMI, functional status, ASA class, and comorbidities
Pulmonary embolism (PE)					
Cloney MB, et al (2018)	30-day PE	Steroid group: 3/353<br>Non-steroid group: 50/8139	1.39 [0.43, 4.47]	NA	NA

Ifarraguerri AM, et al (2023)	30-day PE	Steroid group: 2/178<br>Non-steroid group: 26/10153	4.43 [1.04, 18.79]	4.83 [1.07, 21.89]	Age, sex, BMI, comorbidities, ASA class
Lee R, et al (2020)	30-day PE	Steroid group: 1/198<br>Non-steroid group: 8/5179	3.28 [0.41, 26.36]	15.05 [0.61, 370.72]	Age, sex, smoking, comorbidities, ASA class (Matching)
White SJW, et al (2019a)	30-day PE	Steroid group: 1/289<br>Non-steroid group: 56/9194	0.57 [0.08, 4.11]	NA	NA
White SJW, et al (2019b)	30-day PE	Steroid group: 34/418<br>Non-steroid group: 308/7518	2.07 [1.43, 3.00]	NA	NA
Deep vein thrombosis (DVT)					
Cloney MB, et al (2018)	30-day DVT	Steroid group: 6/353<br>Non-steroid group: 84/8139	1.66 [0.72, 3.82]	NA	NA
Ifarraguerri AM, et al (2023)	30-day DVT	Steroid group: 0/178<br>Non-steroid group: 46/10153	0.61 [0.04, 9.92]	NA	NA
Lee R, et al (2020)	30-day DVT	Steroid group: 1/198<br>Non-steroid group: 16/5179	1.64 [0.22, 12.41]	1.67 [0.17, 16.14]	Age, sex, smoking, comorbidities, ASA class (Matching)
Newton WN, et al (2023)	30-day DVT	Steroid group: 0/93<br>Non-steroid group: 1/1205	4.29 [0.17, 106.14]	NA	NA
White SJW, et al (2019a)	30-day DVT	Steroid group: 26/289<br>Non-steroid group: 84/9194	10.72 [6.79, 16.93]	NA	NA
White SJW, et al (2019b)	30-day DVT	Steroid group: 17/418<br>Non-steroid group: 122/7518	2.57 [1.53, 4.31]	2.10 [1.24, 3.55]	Age, sex, comorbidities, functional status, ASA class

OR, odds ratio; BMI, body mass index; ASA class, American Society of Anesthesiologists Classification; DVT, deep vein thrombosis; PE, pulmonary embolism

Table S5. Subgroup Analysis for Each Outcome Based on Adjustment Methods of Confounding Factors.

Outcome and subgroups	No. of studies	Effect size (OR [95%CI])	I^2 (%)	Test for subgroup differences (*P*)
Mortality				
Overall adjusted analyses	4	1.40 [0.64, 3.09]	52	0.44
Adjusted by matched data	2	2.41 [0.34, 17.24]	73	
Adjusted by multivariate regression	2	1.04 [0.47, 2.32]	35	
Overall complications				
Overall adjusted analyses	3	1.42 [1.16, 1.75]	0	0.65
Adjusted by matched data	1	1.30 [1.08, 1.57]	NA	
Adjusted by multivariate regression	2	1.44 [0.97, 2.14]	45	
Re-admission				
Overall adjusted analyses	5	1.62 [1.48, 1.77]	0	0.14
Adjusted by matched data	3	1.46 [1.25, 1.72]	0	
Adjusted by multivariate regression	2	1.69 [1.52, 1.88]	0	
30-day re-operation				
Overall adjusted analyses	4	1.28 [1.03, 1.59]	32	0.10
Adjusted by matched data	2	1.05 [0.80, 1.37]	0	
Adjusted by multivariate regression	2	1.42 [1.12, 1.81]	13	
Sepsis				
Overall adjusted analyses	4	2.07 [1.34, 3.21]	0	0.94
Adjusted by matched data	2	2.04 [1.10, 3.77]	0	
Adjusted by multivariate regression	2	2.11 [1.14, 3.91]	0	
Deep surgical site infection				
Overall adjusted analyses	4	1.96 [1.26, 3.05]	0	0.63
Adjusted by matched data	2	1.61 [0.59, 4.34]	12	
Adjusted by multivariate regression	2	2.13 [1.19, 3.80]	0	
Thromboembolism events				
Overall adjusted analyses	3	1.61 [0.99, 2.63]	44	0.63
Adjusted by matched data	2	1.50 [0.67, 3.37]	64	
Adjusted by multivariate regression	1	1.92 [1.10, 3.36]	NA	
Pulmonary embolism				
Overall adjusted analyses	2	5.94 [1.52, 23.29]	0	0.53
Adjusted by matched data	1	15.05 [0.61, 370.72]	NA	
Adjusted by multivariate regression	1	4.83 [1.07, 21.89]	NA	
Deep vein thrombosis				
Overall adjusted analyses	2	2.07 [1.24, 3.46]	0	0.85
Adjusted by matched data	1	1.67 [0.17, 16.14]	NA	
Adjusted by multivariate regression	1	2.10 [1.24, 3.55]	NA	
